# The Diagnostic Role of Multimodal Imaging Techniques in Isolated Foveal Hypoplasia

**DOI:** 10.4274/tjo.43799

**Published:** 2017-10-27

**Authors:** Figen Batıoğlu, Sibel Demirel, Emin Özmert, Betül Bayraktutar, Özge Yanık

**Affiliations:** 1 Ankara University Faculty of Medicine, Department of Ophthalmology, Ankara, Turkey

**Keywords:** Foveal hypoplasia, fundus autofluorescence, optical coherence tomography

## Abstract

To report a case of bilateral isolated foveal hypoplasia in which multimodal imaging was used to confirm the diagnosis. Fundus autofluorescence imaging, optical coherence tomography (OCT), and fundus fluorescein angiography were used to describe the typical findings of a patient with isolated foveal hypoplasia. Spectral domain OCT showed absence of foveal depression and persistent inner retinal layers in the fovea. Fundus autofluorescence did not reveal foveal hypoautofluorescence in the presumed foveal area. Clinical diagnosis of foveal hypoplasia may be difficult due to the subtle nature of fundus findings. Fundus autofluorescence imaging may help to diagnose these patients. Foveal hypoplasia should be considered in the differential diagnosis of absence of foveal hypoautofluorescence.

## INTRODUCTION

Isolated foveal hypoplasia (IFH) is a condition in which the fovea is characterized by the absence of foveal depression, pigmentation, and foveal avascular zone.^1,2,3^ It may occur in isolation or in association with conditions such as albinism, aniridia, retinopathy of prematurity, achromatopsia, microphthalmus, myopia, and incontinentia pigmenti.^1,2,3,4,5^ No single hereditary pattern has been established for patients with IFH. Reported cases include patients with autosomal dominant and autosomal recessive inheritance patterns as well as sporadic cases.^1,6^ Some authors described the absence of genes such as *PAX6, OCA2*, and *GPR143*, which are associated with ocular albinism in IFH.^2^

There is wide variability in clinical manifestations of the disease. In most cases, there is decreased visual acuity and an association with nystagmus, and the clinical diagnosis may be difficult due to the subtle nature of fundus findings. Optical coherence tomography (OCT) has been described as a quick and useful tool to confirm the diagnosis of IFH and a grading system based on OCT findings has been developed.^6,7^ Herein, we report a case of bilateral IFH in which multimodal imaging was used to confirm the diagnosis and we describe the fundus autofluorescence (FAF) pattern.

## CASE REPORT

**Figure 1:**
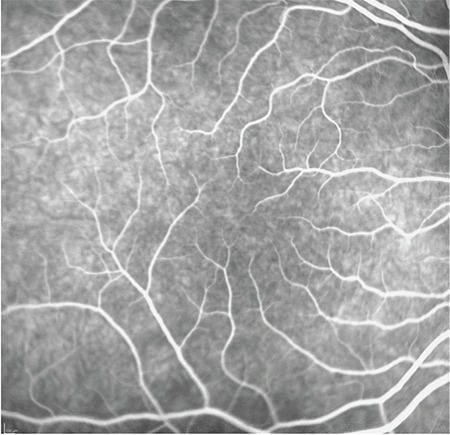
Perifoveal capillaries were abnormally close to the presumed foveal area, with some crossing the horizontal meridian

**Figure 2:**
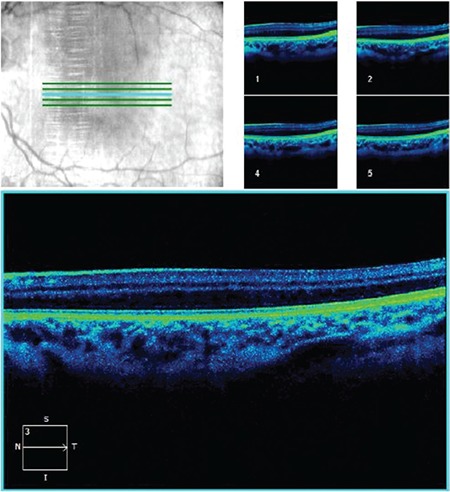
Spectral domain optical coherence tomography showed an absence of foveal depression and persistent inner retinal layers in the fovea

**Figure 3:**
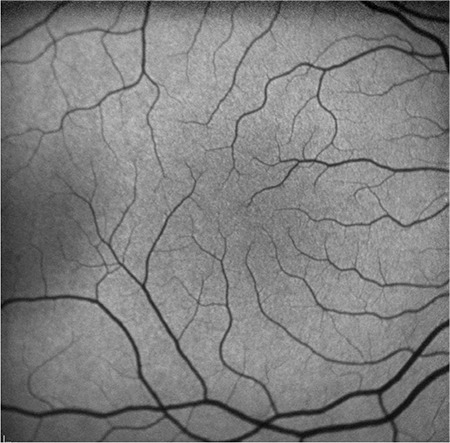
Fundus autofluoresce imaging did not reveal foveal hypoautofluorescence in the presumed foveal area

A 14-year old girl was referred to our department with the complaint of non-progressive reduced vision since childhood. Her family and medical history were unremarkable and she was not born prematurely. The best corrected visual acuity was 0.3 in the right eye and 0.4 in the left eye. There was no nystagmus or iris transillumination suggestive of ocular albinism in either eye. Iris and anterior chamber angle were normal with no sign of aniridia. Fundus examination revealed the absence of foveal reflex and macular pigmentation with normal appearance of optic nerve heads. Fluorescein angiography (FA) revealed the absence of capillary-free zone and the intensity of choroidal fluorescence from the macular area was similar to that from other parts of the retina. In addition, the perifoveal capillaries were abnormally close to the presumed foveal area and some crossed the horizontal meridian (Figure 1). Spectral domain OCT (Cirrus High Definition OCT; Carl-Zeiss Meditec) showed an absence of foveal depression and persistent inner retinal layers in the fovea (Figure 2). It also demonstrated the absence of extrusion of plexiform layers, the absence of outer segment lengthening, and the presence of outer nuclear layer widening corresponding to grade 3 foveal hypoplasia as described by Thomas et al.^7^ FAF imaging did not reveal foveal hypoautofluorescence in the presumed foveal area (Figure 3).

## DISCUSSION

IFH is a rare condition in the absence of other ocular manifestations. In vitro histological examinations of foveal hypoplasia showed that the retina at the posterior pole remained at the stage of differentiation normally exhibited in the sixth month in utero.^5^ Thomas et al.^7^ developed a grading system according to the presence or absence of foveal pit and widening of the outer nuclear layer and lengthening of outer segment at the fovea. The grading system may also show at which stage foveal development was arrested. Our patient exhibited grade 3 foveal hypoplasia according to this system. As reported previously, capillaries which crossed the horizontal meridian and an absence of capillary avascular zone were noted on FA imaging.^1,5,8,9^ However, in contrast to many other case reports, our patient did not present with nystagmus. We used multimodal retinal imaging systems for in vivo confirmation of the diagnosis of foveal hypoplasia.

Previous reports have described a FAF pattern in foveal hypoplasia.^1,9^ In the present case, FAF imaging did not show the typical foveal darkening due to absence of the macular pigments and we observed similar autofluorescence at the macular area compared with peripheral parts of the fundus. We concluded that this phenomenon may be related to the amount of macular pigment in the fovea. This is supported by Charbel Issa et al.,^9^ who reported that the usual foveal attenuation of FAF by macular pigment is reduced in these patients. Mota et al.^1^ also reported a lack of foveal darkening in one patient and only slightly reduced foveal attenuation of autofluorescence in their other foveal hypoplasia cases. However, visual acuity was better in their first patient despite a lack of normal foveal depression as well as lack of foveal darkening on FAF image. In contrast to this report, Charbel Issa et al.^9^ reported that reduced foveal darkening was more pronounced in their second patient, who had worse visual acuity, and they speculated that macular pigment density correlated with the anatomical and functional integrity of the fovea in patients with foveal hypoplasia. In accordance with this previous report, this finding was prominent in our patient, who had low visual acuity and severely disorganized macular anatomy.

Although SD-OCT, a quick and non-invasive method, is helpful in the diagnosis of foveal hypoplasia,^6,7^ especially in patients with decreased visual acuity, the clinical diagnosis may be difficult due to the subtle nature of fundus findings. FAF imaging may also help to diagnose these patients. Foveal hypoplasia should be considered in the differential diagnosis of absence of foveal hypoautofluorescence.
